# The Effect of Cold Ischemia Time and/or Formalin Fixation on Estrogen Receptor, Progesterone Receptor, and Human Epidermal Growth Factor Receptor-2 Results in Breast Carcinoma

**DOI:** 10.1155/2012/947041

**Published:** 2012-03-11

**Authors:** Melike Pekmezci, Anna Szpaderska, Clodia Osipo, Çağatay Erşahin

**Affiliations:** ^1^Department of Pathology, Loyola University Medical Center, Maywood, IL 60153, USA; ^2^Department of Surgery, Loyola University Medical Center, Maywood, IL 60153, USA

## Abstract

*Aims*. To compare the results of estrogen and progesterone receptors (ER, PR), and human epidermal growth factor receptor-2 (HER2) expression status on biopsy and excision specimens and to evaluate the effect of cold ischemia time and/or formalin fixation on these biomarkers. *Methods*. Breast carcinomas that were diagnosed between 2007 and 2009 by core needle biopsy, and subsequently excised in our institution, were included in the study. Data regarding the tumor morphology, grade, and ER, PR, and HER2 status were retrospectively collected from the pathology reports. *Results*. Five out of 149 (3.4%) cases with ER-positive receptor status in the biopsy specimen became ER-negative in the subsequent excision specimen. Nine out of 126 (7.1%) cases with PR-positive receptor status in the biopsy specimen became PR-negative in the excision specimen. Receptor status change was predominantly seen in tumors with low ER and PR receptor expression. HER2 results were consistent between biopsy and excision specimens in all cases tested. *Conclusions*. Cold ischemia time and/or formalin fixation affect mainly ER and PR testing with low Allred scores and support the implementation of the ASCO/CAP guidelines. HER2 results, however, were not affected in our limited number of patients.

## 1. Introduction

 Breast cancer is one of the best examples where antibody-defined tumor markers are used as both prognostic and predictive factors. Prognostic factors are independently associated with the clinical outcome, whereas predictive factors are independently associated with response or lack of response to a particular treatment. Estrogen receptor (ER) expression is a positive prognostic marker of outcome and a strong predictive marker of response to hormone-based therapies such as tamoxifen [1, 2]. Similarly, progesterone receptor (PR) expression is correlated with better prognosis and higher response to hormone-based treatments and increases the predictive power of ER [3–5]. Yet another important marker in the evaluation of breast cancer is the human epidermal growth factor receptor-2 (HER2; c-erbB-2), which is a member of the epidermal growth factor receptor family. HER2 overexpression and/or gene amplification have been shown to be a poor prognostic factor in breast cancer [6, 7]. HER2 status is also predictive for sensitivity to anthracycline-based chemotherapies and relative resistance to cytoxan-based and tamoxifen-based adjuvant therapies [8]. Moreover, it is essential for the therapeutic decisions regarding the use of agents targeting the HER2 gene product such as the humanized, monoclonal antibody, trastuzumab [9].

 The current standard of care for breast cancer requires detection of ER and PR status by immunohistochemistry (IHC) and detection of HER2 status by IHC and/or fluorescence in situ hybridization (FISH). There are several factors that can potentially interfere with the accuracy of results of these tests including tissue fixation (type of fixative, cold ischemia time, and duration of fixation), choice of tissue (core needle biopsy versus excision specimen), choice of IHC assay, and threshold for interpretation of positivity. The American Society of Clinical Oncology and College of American Pathologists (ASCO/CAP) developed guideline recommendations for tumor marker testing in breast cancer based on currently available literature to improve the accuracy and the reproducibility of these tests [10, 11]. In summary, they recommended that core needle biopsies should be preferred for testing if they are representative of the tumor, cold ischemia time should be kept to less than 1 hour, and samples should be fixed in 10% neutral buffered formalin (NBF; formalin in water, 10% by volume, pH 7.4) no less than 6 hours and no more than 72 hours to comply with the panel recommendations for ER and PR testing. Recommended cold ischemia time for HER2 testing is not specifically mentioned but it should be as short as possible, and specimens should be fixed in 10% NBF no less than 6 and no more than 48 hours.

 At our institution, all core needle biopsies have been immediately placed into 10% NBF as a standard procedure (<1 hour) for many years. However, there was no recorded information regarding the cold ischemia time for surgical specimens before the adaptation of ASCO/CAP guideline recommendations. Cold ischemia time is estimated to be more than 1 hour in all specimens. The purpose of this study is to compare the results of ER, PR, and HER2 expression status on biopsy and excision specimens and to evaluate the effect of cold ischemia time and/or formalin fixation on these biomarkers.

## 2. Materials and Methods

 This study was approved by the Loyola University Medical Center (LUMC) Institutional Review Board. We conducted a pathology database search for all patients with in situ or invasive breast carcinomas diagnosed by a core needle biopsy between January 2007 and December 2009. Patients who underwent subsequent tumor excision (excisional biopsy or mastectomy) at LUMC were included in the study. Patients who received treatment between the core needle biopsy and the surgery were excluded from the study. Cases seen in pathology consultation, excision specimens without a biopsy cavity and/or scar, and specimens without diagnostic tissue or without immunohistochemical stains for hormone receptors were also excluded from the study. Data regarding the duration of fixation, tumor morphology, grade, and hormone receptor and HER2 status were retrospectively collected from the pathology reports.

### 2.1. Specimen Collection and Processing

 Core needle biopsies have been routinely placed in 10% NBF at the time of procedure at the Radiology Department. Therefore, cold ischemia time was under 1 hour for all core needle biopsies. Excisional biopsy (lumpectomy) and mastectomy specimens have been received by pathology after the completion of the surgery. There is no record of the time when the specimen was collected from the patient. Hence, cold ischemia time is unknown for surgical specimens and estimated to be more than 1 hour in all specimens. After inking of the margins, specimens were sliced in 0.5 cm thickness and placed in 10% NBF at the Pathology Department. The duration of fixation has been routinely recorded for all specimens and complies with the panel recommendations of 6 to 48 hours.

IHC analysis of ER, PR, and HER2 was performed on the Benchmark XT staining module (Ventana Medical Systems Inc, Tucson, AZ). Paraffin sections were cut at 5 *μ*m and placed on positively charged slides. Slides were incubated in a 70°C oven for 2 hours for ER and PR and air-dried at ambient temperature overnight for HER2. CONFIRM anti-ER (SP1, 1 *μ*g/mL), CONFIRM anti-PR (1E2, 1 *μ*g/mL), and PATHWAY anti-HER2/neu (4B5, 6 *μ*g/mL) rabbit monoclonal antibodies (Ventana Medical Systems Inc) were used as primary antibodies. Deparaffinization, epitope retrieval via cell conditioning (CC1, Ventana) for 90 minutes, antibody incubation at 37°C or 30 minutes, and counterstaining with hematoxylin were performed according to the automated slide stainer protocol. Unstained slides were sent to an outside laboratory (Genzyme) for detection of HER2 gene amplification by FISH.

### 2.2. Interpretation and Reporting of the Results

 H&E and IHC studies were evaluated by one or more of the three experienced breast pathologists. ER, PR, and HER2 staining was assessed according to ASCO/CAP guideline recommendations [10, 11]. In addition to the positive internal controls, an external control (breast tumor with known ER, PR or HER2 positivity, resp.) was evaluated on the same slide with the diagnostic tissue. Allred scores were calculated for ER and PR [2]. HER2 FISH results were provided as negative, equivocal, or positive by the outside lab in concordance with ASCO/CAP guidelines [10].

### 2.3. Statistical Analysis

 Statistical analyses were performed using SPSS 11.0.1 (SPSS Inc., Chicago, IL). Descriptive data were presented as means with standard deviations. Comparisons among the Allred scores of biopsy and excision specimens for ER and PR were performed with Wilcoxon Signed-Rank test. Comparisons among biopsy and excision rates of ER and PR expression were performed with Pearson's chi-square and Fisher's Exact tests. Results with a *P* value less than 0.05 were accepted as significant.

## 3. Results

 We identified 679 patients based on our database search and included 190 patients in the study. Patients who had either biopsy or surgery performed at an outside hospital (*n* = 320), patients with neoadjuvant chemotherapy (*n* = 11) and patients with specimens lacking diagnostic tissue (*n* = 18) or tumor marker studies in both specimens (*n* = 140) were excluded from the study. Mean age of the patients at the time of surgery was 62.2 ± 14.0 years and average size of the tumor based on gross or microscopic evaluation was 2.00 ± 1.70 cm. There were 23 ductal carcinomas in situ (DCIS), 137 invasive ductal carcinomas, 18 invasive lobular carcinomas, and 12 other invasive tumors including invasive solid papillary carcinoma, apocrine carcinoma, and metaplastic carcinoma. The duration of fixation complies with the ASCO/CAP guideline recommendations for all specimens; 8.7 ± 3.3 (median: 8, range: 6–34) hours for biopsies and 22.2 ± 9.2 (median: 26, range: 6–48) hours for excision specimens (*P* < 0.001). The duration of fixation was similar between ER-positive and ER-negative biopsies, ER-positive and ER-negative excisions, as well as PR-positive and PR-negative biopsies (data not shown; *P* > 0.05 for all). The duration of fixation was slightly longer (25.5 ± 8.7 hours) for PR-positive excisions as compared to PR-negative excisions (21.7 ± 9.8 hours; *P* = 0.022).

**Figure 1:**
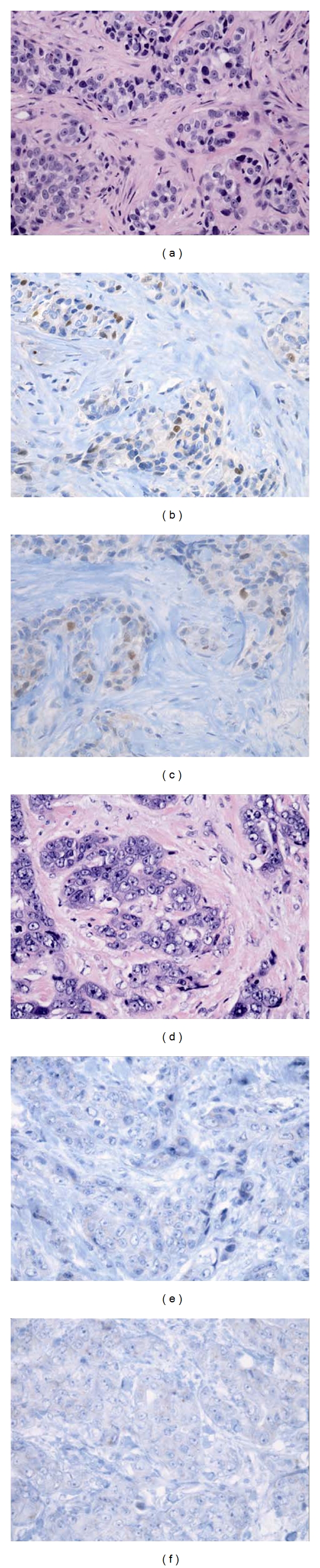
Histology and hormone receptor staining of a case with estrogen-receptor (ER-) positive, progesterone-receptor- (PR-) positive results in biopsy and ER-negative, PR-negative results in subsequent excision. (a) Biopsy, invasive ductal carcinoma, Nottingham Grade III, hematoxylin & eosin (400x); (b) biopsy, ER (+), Allred score 3 (400x); (c) biopsy, PR (+), Allred score 3 (400x); (d) excision, invasive ductal carcinoma, Nottingham Grade III, hematoxylin & eosin (400x); (e) excision, ER (−), Allred score 0 (400x); (f) excision, PR (−), Allred score 0 (400x).

 ER status was evaluated in all biopsies and 149 out of 190 (78.4%) were positive (Tables 1 and 2). ER status was evaluated in all excision specimens and 144 out of 190 (75.9%) were positive. Five out of 149 (3.4%) cases with ER-positive receptor status in the initial biopsy specimen became ER-negative in the subsequent excision specimen (Figure 1). Negative staining was verified with a second study in all cases. The false-negative rate for the ER receptor on the excision specimen was 10.9% (*P* < 0.001). All the cases that converted from ER-positive to ER-negative had an Allred score of 3 with a positivity ratio of 1%. Allred scores of ER-positive receptor status in biopsies that remained positive in the excision specimens had an Allred score of 5 and higher. The average Allred score for ER was 6.1 ± 3.3 among biopsy specimens and 5.9 ± 3.4 among excision specimens (*P* = 0.004).

 PR status was evaluated in 186 out of 190 biopsies (97.9%), and 126 out of 186 (67.7%) were positive (Tables 1 and 3). PR status was evaluated in 189 out of 190 excision specimens (99.5%), and 123 out of 189 (65.1%) were positive. There were 14 discrepant results for PR receptors between the biopsy and excision specimens of the same tumor. Five biopsy cases with negative PR receptors were reported to be PR-positive in the excision specimen. Nine out of 126 (7.1%) cases with PR-positive receptor status in the biopsy specimen became PR-negative in the subsequent excision specimen. Negative staining was verified with a repeat study in three cases. The false-negative rate for PR receptors on the excision specimen was 14.1% (*P* < 0.001). The cases that converted from PR-positive in biopsies to PR-negative in excision specimens had a lower Allred score of 4.3 ± 1.9 as compared to other cases that remained positive (7.0 ± 1.3; *P* < 0.001). The average Allred score for PR was 4.6 ± 3.4 among biopsy specimens and 4.5 ± 3.4 among excisions (*P* > 0.05).

 Out of five cases that converted from ER-positive in biopsies to ER-negative in excision specimens, two had PR-negative receptors both in biopsies and excisions, two had PR-positive receptors in biopsy specimens that converted to PR-negative in excision specimens, and one had PR-positive receptors in the biopsy specimen that remained PR-positive in the excision specimen. The latter was the only specimen in our series with an ER-negative, PR-positive result.

 Among the cases with invasive tumors (*n* = 167), an IHC evaluation of HER2 status was performed in 164 (98.2%) biopsies. Among these, 15 were positive (3+), 32 were equivocal (2+), and 117 were negative (0 or 1+). Only 19 out of 32 equivocal biopsies had further testing with FISH, and, out of 19, only one (5.3%) tested positive. Overall, 16 (9.8%) biopsies were classified as positive, 13 (7.9%) biopsies were classified as equivocal, and 135 (82.3%) were classified as negative after FISH evaluation. IHC evaluation of HER2 status was possible in all excision specimens and, among these, 16 (9.6%) were positive, 123 (73.7%) were negative, and 28 (16.8%) were equivocal. Twenty-five out of 28 equivocal specimens were further evaluated by FISH and two (8%) tested positive. Therefore, after FISH evaluation, 18 (10.8%) were classified as positive, 3 (1.8%) were classified as equivocal, and 146 (87.4%) were classified as negative. Three cases without HER2 evaluation in biopsy specimens had negative IHC results in their excision specimens. There was no discrepancy between the IHC and FISH results for both the biopsy and excision specimens.

 Based on the final classification (considering both IHC and FISH), there was no clinically significant discrepancy for HER2 status between the biopsy and the excision specimens of the same tumor. Exact concordance was seen in 146 (89%) out of 164 cases. There was one case with an equivocal IHC result in biopsy and a positive IHC result in excision; however, FISH analysis of the biopsy was positive and this case was not considered discrepant. There were two equivocal HER2 status in biopsies (equivocal IHC staining, no FISH evaluation) later classified as positive (equivocal IHC staining, positive FISH) in the excision specimens, which were not considered discrepant. There was no discrepancy between the FISH results of biopsies and excisions.

## 4. Discussion

 ER, PR, and HER2 expression status of a breast cancer has significant prognostic and predictive value. Hence, invalid test results could significantly change the therapeutic management of a patient with potentially negative effects on the outcome. Along with analytic (choice of assay) and postanalytic (choice of cutoffs) factors, preanalytic factors play a significant role in the accuracy and precision of these tests. All steps of specimen handling, including cold ischemia time, duration of fixation, and type of fixative, have an impact on the result, and optimization of tissue handling is essential for clinical utility of these tests. At our institution, all core needle biopsy materials have been directly placed into 10% NBF after acquiring and, therefore, cold ischemia time for those specimens has been minimal. In contrast, cold ischemia time for the surgical excision specimens has not been recorded before the adaptation of ASCO/CAP guideline recommendations for specimen handling for hormone receptor testing in breast cancer and was estimated to be more than 1 hour in almost all specimens. In this study, we compared surgically excised tumors with the preceding core needle biopsies from the same tumor for ER, PR, and HER2 status to evaluate the effects of cold ischemia time and/or formalin fixation.

 We identified 5 out of 149 patients whose tumors that were initially ER-positive in core needle biopsies later became ER-negative by IHC in their excision specimen. Similarly, 9 out of 126 patients whose tumors that were PR-positive in biopsies later became PR-negative by IHC in their excision specimen. With more than 10% false-negative rates, these results are both statistically and clinically significant. According to current treatment algorithms, these patients would be inappropriately denied hormone-based chemotherapies, and their prognoses would be negatively affected if treated based only on excision specimen results.

 Our false-negative results are similar the previous studies in the literature [12, 13]. A recent study by Uy et al. reported that 25 out of 152 ER-positive core biopsies and 17 out of 150 PR-positive core biopsies became ER- and PR-negative in the mastectomy specimen, respectively [12]. In another study, Mann et al. reported that concordance rates for ER and PR status between core biopsy and surgical specimens were 86% and 83%, respectively [13]. Respective false-negative rates for ER and PR on surgical specimens in their series of 100 patients were 14% and 15%.

 Our results also showed that Allred scores for ER on biopsy specimens were significantly higher than those on excision specimens. This finding agrees with the previous studies reporting higher rate of ER staining and Allred scores on core and incisional biopsy specimens [12–16]. This can be explained by loss of hormone receptors secondary to various factors including longer warm and cold ischemia times, insufficient fixation due to larger size of mastectomies and/or tumor heterogeneity [14, 15, 17–21]. We could not show a significant difference between the biopsy and excision specimens for PR Allred scores. However, this analysis could be affected by the cases with PR-negative biopsies and corresponding PR-positive excisions. We believe that the initial PR-negativity of these biopsies was most likely due to sampling. Sampling has been described as a significant factor for false-negative PR results in small biopsies due to more heterogeneous PR expression of the tumor cells [22].

 Higher Allred scores and percentages of staining in biopsy specimens can be explained by better preservation of the receptor proteins with timely fixation. Delayed formalin fixation and associated long ischemia times were reported to be negatively correlated with the hormone receptor expression in the diagnostic specimens [18, 20, 21]. A decreased protein level by delayed fixation was initially shown by ligand binding assays [18]. Recently, an experimental study on 10 breast cancer cases reported that a progressive delay of fixation was correlated with a progressive decrease of both the percentage and intensity of ER, PR, and HER2 IHC staining of tumor cells [20]. Although their numbers were limited to reach statistical significance, they have one case that showed conversion of positive staining in immediately fixed sample to negative staining in late-fixed samples. The same group further reported that the negative impact of delayed formalin fixation is independent of the antibody clone used for the testing [21]. Another experimental study reported no change in the ER and PR results of a strongly and diffusely ER-positive, PR-positive breast carcinoma after storage at 4°C for four days. However, as they have also mentioned in their discussion, this finding may not be extrapolated to the tumors with weak ER and PR positivity [23].

 Our results showed that cases with false-negative IHC results on excision specimens had lower Allred scores in biopsy specimens for both ER and PR. All ER-positive biopsies that became ER-negative in the excision had an Allred score of 3, with weak staining of 1% of the tumor cells. Similarly, Allred scores of PR-positive biopsies that became PR-negative in the excision specimen were significantly lower than those that remained positive. Because these cases had only few positive receptors, it is expected that they are particularly at risk for false negativity. To our knowledge, this is the first study comparing the effect of cold ischemia time on tumor markers with low and high Allred scores in real patient data.

 In our study, there was no clinically significant discrepancy between biopsies and excision specimens regarding the HER2 receptor results as assessed by the combination of IHC and FISH. There were 10 cases with HER2-equivocal results in biopsy and HER2-negative results in subsequent excision specimens. Seven cases had HER2-negative results in biopsy and HER2-equivocal results in excision specimens, and 1 case had HER2-equivocal result in biopsy and HER2-positive result in the excision specimen. There was no discrepancy when the equivocal cases were reclassified via FISH results except for the cases without FISH analysis. Nevertheless, the possibility of the effect of delayed fixation on HER2 results cannot be entirely ruled out due to the low number of cases. A previous study comparing the results of core and incisional biopsies reported a concordance rate of 80% [13]. Seventeen out of 20 discrepant cases had HER2-equivocal core biopsy and HER2-negative surgical specimens. Two cases had HER2-negative biopsy and HER2-equivocal surgical specimens and one case had HER2-positive core biopsy and HER2-negative surgical specimen. Without further FISH analysis, the clinical significance of conversion from equivocal to negative IHC is not clear. Another study evaluating the IHC and FISH results of the tumor samples that were placed in a fixative at different time intervals reported variable and inconsistent IHC results in addition to higher numbers of compromised FISH results with longer cold ischemia times [20]. Two recent studies reported that cold ischemia time has no effect on HER2 FISH results [24, 25].

Our study has some limitations due to its retrospective nature. We had to exclude some cases wherein biopsy or surgery was performed at an outside hospital, patients underwent neoadjuvant chemotherapy, and/or there was an absence of diagnostic tissue or tumor marker studies in both specimens. Not all cases had repeat IHC and FISH studies on both biopsy and excision specimens, which might induce a selection bias in our study population. We do not have accurate data about the cold ischemia time for excision specimens. Although we had data documenting the time that the specimen was received by the pathology department, we have no way of tracking the time of collection from the patient retrospectively. However, we strongly believe that cold ischemia time was more than 1 hour in all excision specimens. Typically there are no weekend specimens in our institution.

Formalin fixation is expected to be slower in surgical excision specimens than it is in core needle biopsies due to the size of the specimens. Although surgical specimens were sliced before formalin fixation, sections are thicker (0.5 cm versus 0.2 to 0.3 cm) and expected to be fixed less efficiently compared to biopsy specimens.

Formalin fixation times were longer for excision specimens compared to biopsies, although both groups were compliant with the current ASCO/CAP guidelines. We believe that longer fixation of larger surgical specimens might have reduced the number of discrepant cases in our study. Although ASCO/CAP guidelines provide the minimum and maximum fixation times, further studies are required to assess the “optimum” fixation times for certain types or sizes of specimens.

 Tumor heterogeneity is a potential problem with the assessment of IHC staining in only one section both in biopsy and excision specimens. We routinely use the largest tumor section for IHC studies and cannot entirely rule out the possibility of a false negative result due to tumor heterogeneity in specimens.

ASCO/CAP guidelines do not recommend the storage of slides for more than 6 weeks before analysis. Disadvantages of archived unstained slides for IHC studies including ER, PR, and HER2 were previously described by multiple studies [26, 27]. Our stains were performed and interpreted at the time of original diagnosis. Therefore, our results are free of any impact of storage of paraffin blocks and slides.

 Accurate assessment of ER, PR, and HER2 status of breast cancers is critical for the correct assignment of the chemotherapeutic regimen. This is also important for the validity of the clinical studies comparing the therapeutic efficacy of various agents among receptor positive and negative tumors. Multiple factors starting with the delayed fixation might indeed explain reports of hormone receptor-negative tumors responding to hormone-based chemotherapies and HER2-negative tumors responding to trastuzumab treatment [28, 29].

 Our results show that cold ischemia time and/or formalin fixation predominantly affect tumors with low ER and PR receptor expression and support ASCO/CAP guideline recommendations including the cold ischemia time being less than 1 hour. We believe that these findings have implications for standardization of clinical practices during the evaluation of the hormone receptor status. Given the importance of the accuracy of these tests, all factors that might cause variation of the results should be clearly listed in the final pathology report and considered during the decision of chemotherapy. There are no studies regarding the best approach to ER, PR, and/or HER2 negative tumors when guideline recommendations were not followed during the handling of the specimen. These results strongly support further studies for evaluation of such tumors in order to understand clinical implications of possible false-negative results and the critical future management strategies.

## Figures and Tables

**Table 1:** Hormone receptor status of breast cancers in core needle biopsy and excision specimens.

			Discrepancy	
	Biopsy	Excision	Biopsy (+) Excision (−)	Biopsy (−) Excision (+)
ER (positive/tested)	149/190	144/190	5	0
PR (positive/tested)	126/186	123/189	9	5

ER: estrogen receptor, PR: progesterone receptor.

**Table 2:** Expression of estrogen receptors in core needle biopsy and excision specimens.

			Biopsy	
		Positive	Negative	Total
Excision	Positive	144	0	144
	Negative	5	41	46
	Total	149	41	190

**Table 3:** Expression of progesterone receptors in core needle biopsy and excision specimens.

			Biopsy	
		Positive	Negative	Total
Excision	Positive	117	5	122
	Negative	9	55	64
	Total	126	60	186
